# Synthesis and acetone sensing properties of ZnFe_2_O_4_/rGO gas sensors

**DOI:** 10.3762/bjnano.10.242

**Published:** 2019-12-16

**Authors:** Kaidi Wu, Yifan Luo, Ying Li, Chao Zhang

**Affiliations:** 1 College of Mechanical Engineering, Yangzhou University, Yangzhou 225127, P.R. China; 2 College of Hydraulic Science and Engineering, Yangzhou University, Yangzhou 225009, P.R. China

**Keywords:** acetone, composites, gas sensor, reduced graphene oxide (rGO), ZnFe_2_O_4_ hollow spheres

## Abstract

Hollow spheres of pure ZnFe_2_O_4_ and of composites of ZnFe_2_O_4_ and reduced graphene oxide (rGO) with different rGO content were prepared via a simple solvothermal method followed by a high-temperature annealing process in an inert atmosphere. The X-ray diffraction analysis confirmed that the introduction of rGO had no effect on the spinel structure of ZnFe_2_O_4_. In addition, the results of field-emission scanning electron microscopy and (high-resolution) transmission electron microscopy indicated that the synthesized samples had the structure of hollow spheres distributed uniformly onto rGO nanosheets. The diameters of the spheres were determined as about 600–1000 nm. The gas sensing test revealed that the introduction of rGO improved the performance of the sensing of acetone to low concentration, and the ZnFe_2_O_4_/rGO composite gas sensor containing 0.5 wt % of rGO exhibited a high sensitivity in sensing test using 0.8–100 ppm acetone at 200 °C. The response of the 0.5 wt % ZnFe_2_O_4_/rGO sensor to 0.8 ppm acetone was 1.50, and its response to 10 ppm acetone was 8.18, which is around 2.6 times more pronounced than the response of pure ZnFe_2_O_4_ (10 ppm, 3.20). Moreover, the sensor showed a wide linear range and good selectivity.

## Introduction

As a synthetic raw material in industrial production, acetone is chemically active and extremely flammable. It is toxic if its concentration exceeds 173 ppm, and long-term exposure to acetone poses a serious threat to human health [1–2]. Furthermore, acetone is also a fat metabolite in the human body. According to the related literature, the concentration of acetone in the exhaled gas of healthy people is less than 0.8 ppm, while that in exhaled gas of diabetic patients is higher than 1.8 ppm [3–4]. In this view, it may be possible to diagnose diabetes using a nondestructive testing technology based on sensing acetone. Thus, it is necessary to develop novel micro/nanomaterials, which can be applied as high-performance gas sensors to detect acetone at low concentration or to monitor variations of its concentration.

Due to their excellent properties and cost efficiency, gas sensors based on metal oxide semiconductors, such as ZnO [5], SnO_2_ [6], WO_3_ [7], TiO_2_ [8], Er-SnO_2_ [9], Au-In_2_O_3_ [10], GO-WO_3_ [11] and Ni-SnO_2_/G [12] have been widely studied until now. However, their sensing properties regarding low amounts of acetone still need to be enhanced. As a dual metal oxide, AB_2_O_4_ spinel materials received much attention in the field of gas sensing [13–14]. With a unique spinel structure and a narrow bandgap width (≈1.94 eV), zinc ferrite (ZnFe_2_O_4_) has remarkable properties and shows good potential in the field of gas sensing. It was reported that small, well-dispersed ZnFe_2_O_4_ nanoparticles showed a good selectivity to acetone at 200 °C, but the detection limit was only 5 ppm [15]. Porous ZnFe_2_O_4_ double-shell microspheres showed a response to acetone at 206 °C, which is mainly ascribed to their unique morphology [16]. Some researchers also found that Ag-activated hollow spheres of ZnFe_2_O_4_ exhibited an excellent acetone gas-sensing performance at 175 °C [17]. Moreover, ZnFe_2_O_4_/ZnO composites showed an excellent response and recovery performance, which was attributed to their nanostructure and synergistic effects in the heterostructures. This will enable corresponding gas sensors to accurately detect and monitor acetone vapor in real-time. In this view, compounding with certain organic or inorganic material could improve the gas sensing properties of ZnFe_2_O_4_ [18–19].

As a novel 2D carbon-based material, graphene has a unique structure and distinguished properties. Plenty of works are in progress to investigate the applications of graphene or its derivatives in the field of gas sensing [20–21], including room temperature CO_2_ gas sensors and room temperature double-layer graphene NO_2_ gas sensors prepared by deposition processes [22–23]. Furthermore, the combination of metal oxides with graphene or its derivatives can enhance the gas sensing capability by improving the adsorption/desorption ability of the incorporated molecules, the transfer of carriers and the formation of local heterojunctions [24–28]. An optimum ratio of the composition and the fine nanostructure will contribute to obtaining better gas-sensing properties. A gas sensor with 3 wt % reduced graphene oxide (rGO) incorporated into In_2_O_3_ showed a rapid response, an improved stability and a low limit of detection of NO_2_ (10 ppb) [29]. ZnO_1−_*_x_*/rGO composites with 2 wt % rGO had enhanced gas sensing properties compared with pure ZnO, as indicated by an enhanced sensitivity and an improved response/recovery speed [30]. It has been proved that coupling or compounding metal oxides with graphene enhances the electronic characteristics and the gas sensing properties. Furthermore, 0.125 wt % graphene-ZnFe_2_O_4_ was prepared by a solvothermal method, and the corresponding gas sensor exhibited a fine response to 10–100 ppm acetone at 275 °C [31]. A hybrid sensor made of ZnFe_2_O_4_/graphene quantum dots showed a fine sensing response to acetone at low temperature, even at room temperature [32].

Hence, with the aim to fabricate a high-performance acetone gas sensor, we prepared hollow spheres of ZnFe_2_O_4_ and ZnFe_2_O_4_/rGO composites using a one-pot solvothermal method followed by a high-temperature heat treatment process in an inert atmosphere. The nanostructure, the micromorphology and the acetone sensing performance of all samples were discussed. Moreover, the optimized rGO mixing ratio and the operating temperature have been determined as 0.5 wt % and 200 °C, respectively.

## Experimental

### Materials

Aqueous dispersion solution of single-layer graphene oxide (GO) particles with average diameters of less than 500 nm and thicknesses of 0.8–1.2 nm (purity: 99%, concentration: 0.5 mg/mL) was purchased from Nanjing XFNANO Materials Technology Co., Ltd (Nanjing, China). Isopropanol (analytical degree) was obtained from Jiangsu Qiangsheng Functional Chemistry Co., Ltd (Suzhou, China). All other reagents (analytical degree) were purchased from Sinopharm Chemical Reagent Co., Ltd (Shanghai, China).

### Samples synthesis

Hollow spheres of pure ZnFe_2_O_4_ and ZnFe_2_O_4_/rGO composites were prepared via a simple solvothermal method followed by a high-temperature heat treatment process in an inert atmosphere. Firstly, the aqueous dispersion solution of GO (0.5 mg/mL) was further treated with ultrasound for 2 h. Then, different amounts of the GO solution (0, 0.242, 0.602, 1.205 and 2.410 mL) were added to the homogeneous solution of isopropanol (30 mL) and glycerol (8 mL) under slow stirring. Secondly, 0.1098 g Zn(CH_3_COO)_2_·2H_2_O and 0.4042 g Fe(NO_3_)_3_·9H_2_O were dissolved in the obtained homogeneous solution under magnetic stirring for 1 h. Subsequently, the mixed solutions were transferred into a Teflon-lined stainless-steel autoclave (50 mL), and then maintained at 180 °C for 12 h. After natural cooling to room temperature, the obtained suspensions were four times centrifuged using deionized water and anhydrous ethanol and dried at 75 °C for 12 h. Ultimately, these precursors were placed in an argon atmosphere, annealed at 400 °C for 2 h with a heating rate of 5 °C/min to reduce GO to rGO [33–34], and five samples were obtained: hollow spheres of pure ZnFe_2_O_4_ and ZnFe_2_O_4_/rGO composite spheres with 0.1, 0.25, 0.5 and 1 wt % of rGO.

### Characterization methods

The crystal phases of pure ZnFe_2_O_4_ and the ZnFe_2_O_4_/rGO composites were characterized by X-ray diffraction (XRD, Bruker D8 Advance) using Cu Kα radiation at room temperature. The 2θ range was 10−80°, and the scanning rate was 5°·min^−1^. The microscopic morphology and the size of all samples were observed using a field-emission scanning electron microscope equipped with an energy-dispersive spectrometer (FESEM, Hitachi S4800). The nanostructure of the products was examined by transmission electron microscopy (TEM, JEM-2100). High-resolution TEM (HRTEM) and energy-dispersive X-ray (EDX) elemental mappings were recorded using a field-emission transmission electron microscope (Tecnai G2 F30 S-TWIN, FEI).

### Fabrication and sensing test of gas sensors

**Figure 1:**
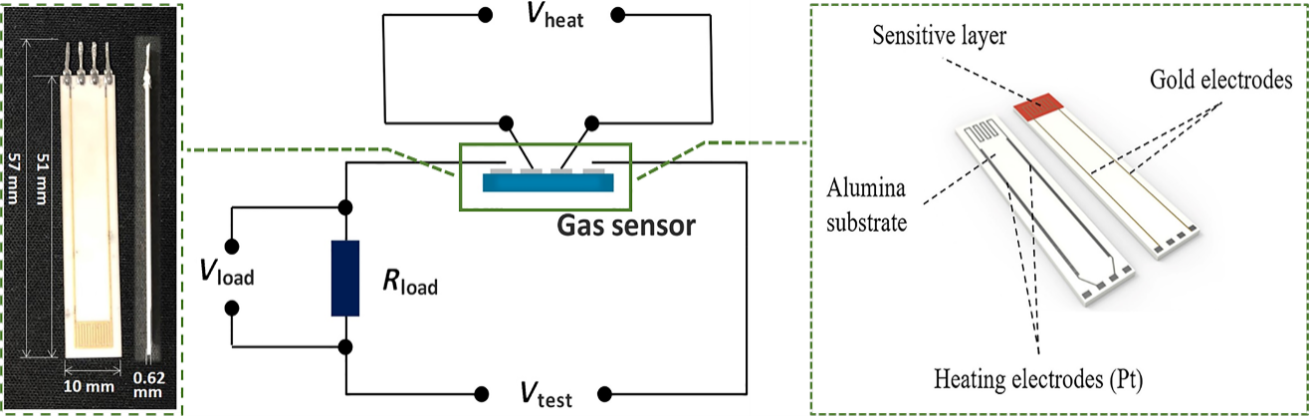
Schematic images of the electric circuit equivalent to the system and the corresponding ZnFe_2_O_4_/rGO sensor.

The hollow spheres of pure ZnFe_2_O_4_ or ZnFe_2_O_4_/rGO powder were mixed with deionized water to obtain a paste, which was then manually uniformly coated onto an Al_2_O_3_ ceramic plate (C-MAC Micro Technology Company, Belgium) equipped with heating electrodes (Pt) and gold electrodes (Au) to fabricate sensing films. Subsequently, the sensors were dried at 120 °C for 12 h, and after further aging for 24 h at 180 °C, a series of ZnFe_2_O_4_/rGO gas sensors (different mass content of rGO: 0, 0.1, 0.25, 0.5 and 1 wt %) were obtained. Figure 1 is the schematic image of an electric circuit equivalent to the sensor system along with the dimensions of the corresponding ZnFe_2_O_4_/rGO gas sensor. The operating temperature of the sensors was adjusted by means of the applied voltage. Further details of the testing system were shown in our previous works [17,30]. The target gas was collected in a poly(methyl methacrylate) (PMMA) chamber (volume: 50 L), the volume of which was calibrated using the following Equation 1:

[1]
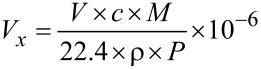


*V_x_* is the volume of the liquid (μL), *c* is the concentration of the gas to be allocated (ppm), *V* is the volume of the collection vessel (mL), *M* is the molecular weight of the substance (g), *P* is the purity of the liquid and ρ is the density of the liquid (g/cm^3^). In addition, the response of the sensors was defined as *S* = *R*_a_/*R*_g_, where *R*_a_ and *R*_g_ are the resistance values of the sensors in air and in test gas, respectively. The response/recovery time is defined as the time required for a change of the resistance of 90%.

## Results and Discussion

### Samples characterization

**Figure 2:**
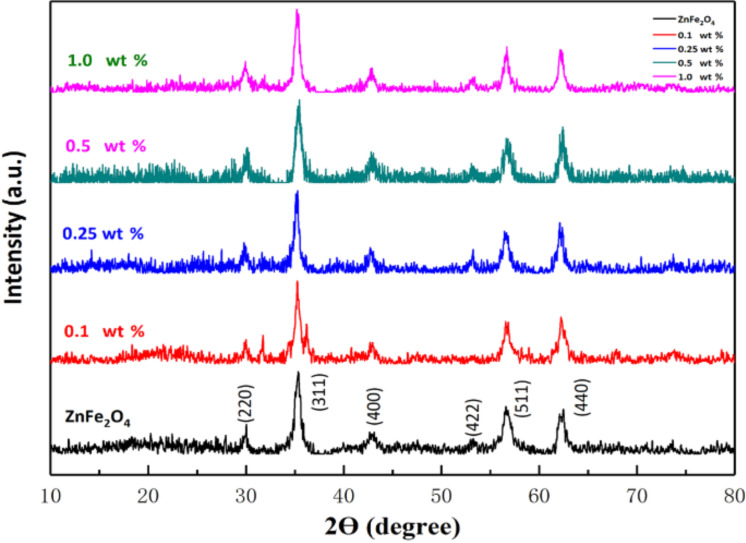
XRD patterns of the synthesized hollow spheres of pure ZnFe_2_O_4_ and the different ZnFe_2_O_4_/rGO composites.

The XRD patterns of the spheres of pure ZnFe_2_O_4_ and the four ZnFe_2_O_4_/rGO composites with different mass fractions of rGO are shown in Figure 2. All samples showed similar diffraction peaks, and according to the well-known diffraction pattern of ZnFe_2_O_4_ (JCPDS Card No. 22-1012), all the peaks of the five samples agree well with the spinel ZnFe_2_O_4_ structure. Hence, the introduction of rGO did not affect the spinel structure of ZnFe_2_O_4_. The characteristic peaks observed at 29.9°, 35.1°, 42.8°, 53.3°, 56.5° and 62.2° were attributed to the (220), (311), (400), (422), (511) and (440) crystal planes, respectively. Furthermore, the strong intensity of the diffraction peaks suggests that both the hollow spheres of pure ZnFe_2_O_4_ and of the ZnFe_2_O_4_/rGO composites were well crystallized. In addition, the characteristic peaks of rGO, that should be observed at about 24°, are not clearly identified in the patterns, which may be ascribed to the low mass fraction of rGO in the ZnFe_2_O_4_/rGO composites [35–36].

**Figure 3:**
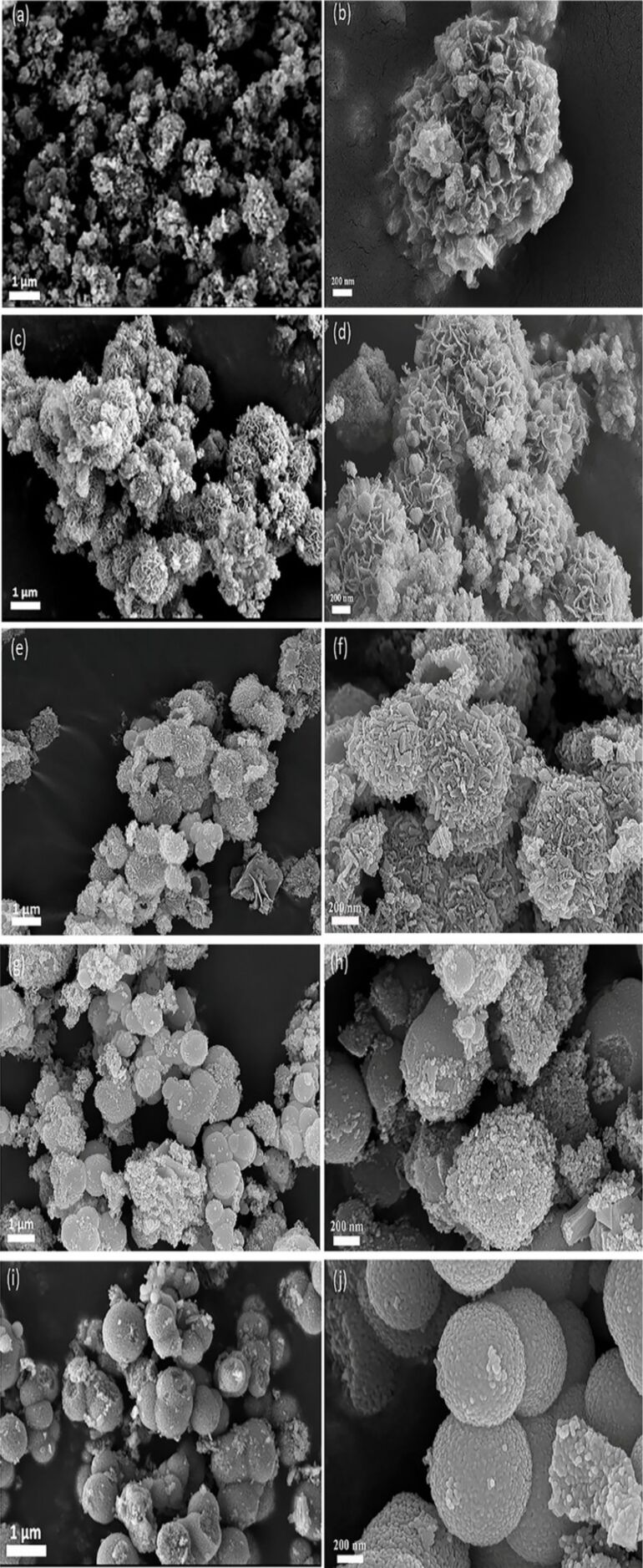
FESEM images of the hollow spheres made of (a, b) pure ZnFe_2_O_4_ and composite ZnFe_2_O_4_/rGO with an rGO percentage of (c, d) 0.1, (e, f) 0.25, (g, h) 0.5 and (i, j) 1 wt %.

The surface morphology and the size of the hollow spheres of pure ZnFe_2_O_4_ and the ZnFe_2_O_4_/rGO composites with different rGO content were analyzed. Figure 3a and Figure 3b show that pristine particles in ZnFe_2_O_4_ powder have a spherical morphology. The mean diameters of the spheres were measured as 600–1000 nm using the ImageJ software. It is observed that the spheres of pure ZnFe_2_O_4_ are composed of small nanosheets. Furthermore, as shown in Figure 3, there is almost no change in the diameter of the ZnFe_2_O_4_/rGO composites when the rGO content is increased from 0 to 1 wt %. However, Figure 3d, Figure 3f, Figure 3h and Figure 3j show that when the rGO content exceeds 0.25 wt %, there are more ZnFe_2_O_4_ spheres which are composed of small nanoparticles instead of nanosheets. This deformation of the self-assembled structure of the nanosheets may be ascribed to the introduction of more water to the organic solvent when increasing the volume of the aqueous GO dispersion [37–38] or to the low mass fraction of rGO in the ZnFe_2_O_4_/rGO composites [17,39].

**Figure 4:**
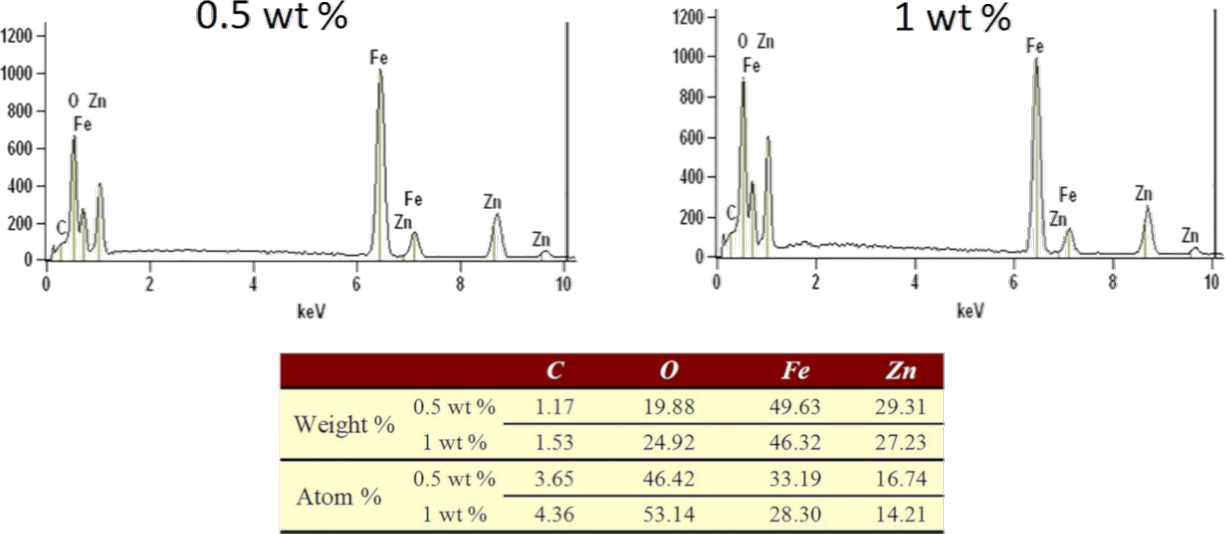
EDX results of the ZnFe_2_O_4_/rGO samples containing 0.5 and 1 wt % rGO.

We analyzed the actual composition of the spheres by energy-dispersive X-ray spectroscopy (EDS). As shown in Figure 4, the weight percentage of carbon in the spheres of ZnFe_2_O_4_/rGO with 0.5 and 1 wt % rGO was determined as 1.17 and 1.53%, respectively. The obtained percentage is higher than theoretically predicted, which may be due to a contamination arising from the carbon-containing electrically conductive adhesive or another source of carbon in the sample stage or on the sample surface. Still, the actual quantities derived by EDX are basically consistent with the theoretical quantities.

**Figure 5:**
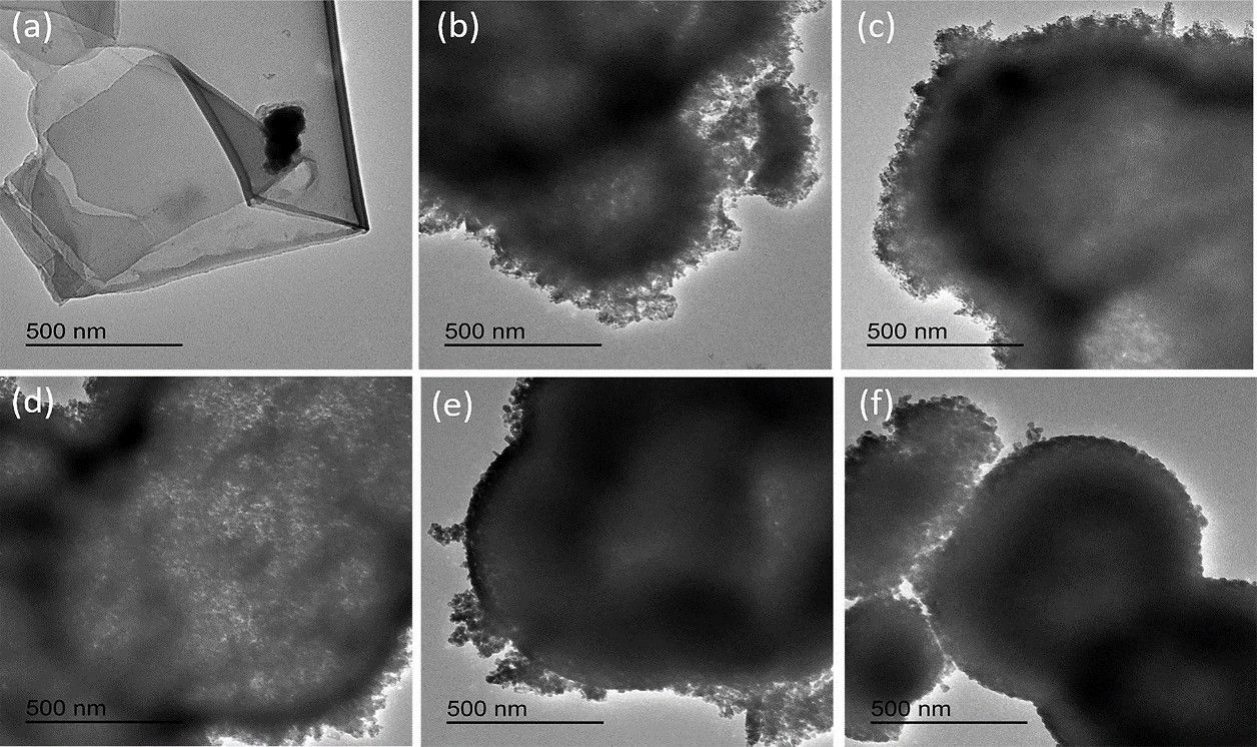
TEM images of (a) GO (b) hollow spheres of ZnFe_2_O_4_ and (c–f) hollow spheres of the ZnFe_2_O_4_/rGO composites containing 0.1, 0.25, 0.5, 1 wt % of rGO.

Figure 5 shows the TEM images of GO and the hollow spheres of pure ZnFe_2_O_4_ and the four ZnFe_2_O_4_/rGO composites. The wrinkles in Figure 5a indicate the two-dimensional structure of GO. Figure 5b–f further shows that the samples made of pure ZnFe_2_O_4_ and ZnFe_2_O_4_/rGO all have the structure of hollow spheres. Furthermore, with increasing rGO content in the ZnFe_2_O_4_/rGO nanomaterial, the surface of the ZnFe_2_O_4_ spheres becomes more regular and smooth, which is consistent with the FESEM images.

**Figure 6:**
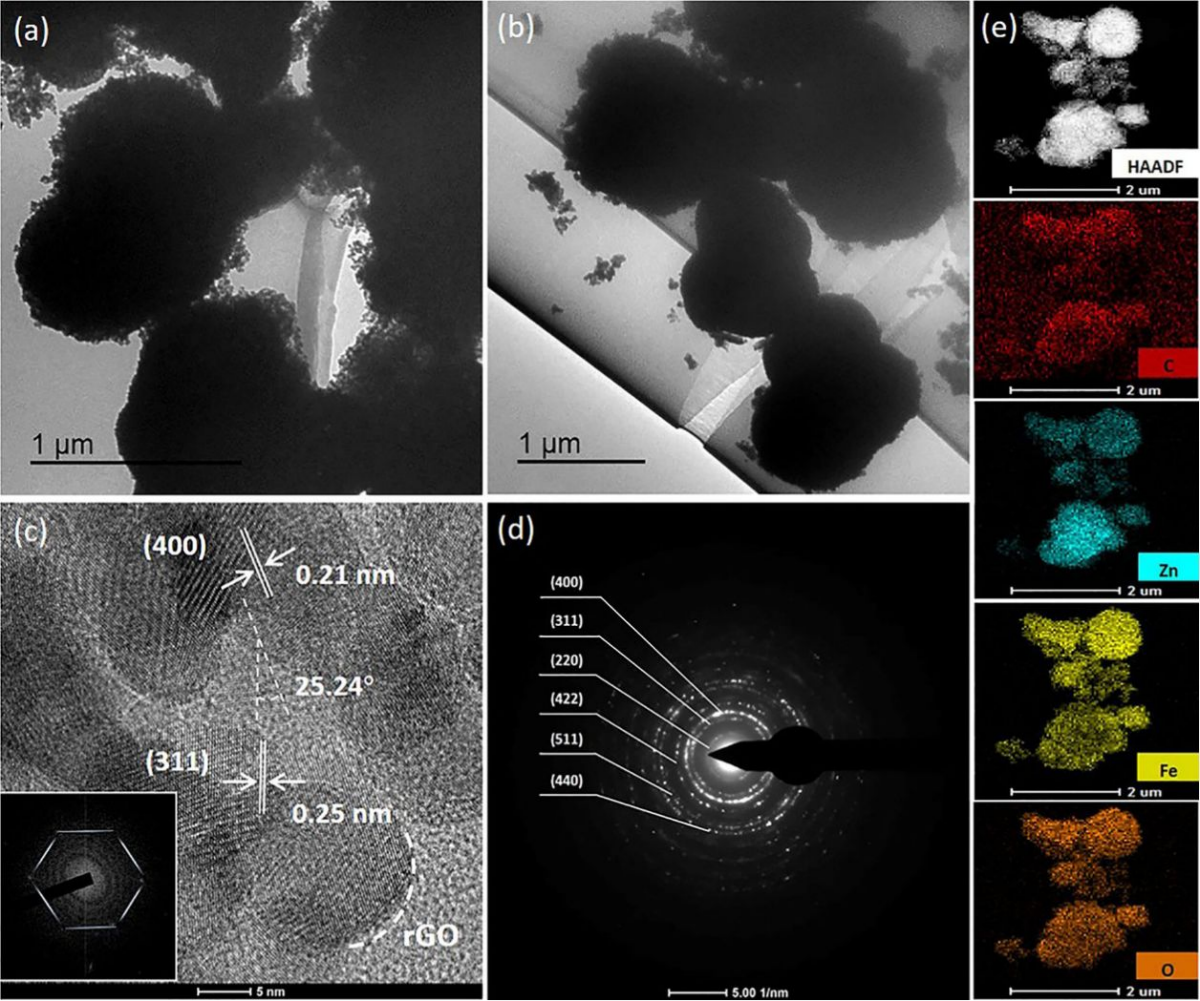
TEM images of (a) the 0.5 wt % ZnFe_2_O_4_/rGO spheres and (b) the 1 wt % ZnFe_2_O_4_/rGO spheres. (c) HRTEM image of the 0.5 wt % ZnFe_2_O_4_/rGO sample, the corresponding FFT image is shown in the inset. (d) SAED pattern of the 0.5 wt % ZnFe_2_O_4_/rGO sample and (e) HAADF-STEM image of the 0.5 wt % ZnFe_2_O_4_/rGO spheres and e) EDX elemental mapping images of C (red), Zn (blue), Fe (yellow) and O (orange).

A further analysis of the nanostructure of the ZnFe_2_O_4_/rGO sample with 0.5 wt % rGO was carried out using TEM and HRTEM (Figure 6). As obvious from Figure 6a and Figure 6b, the ZnFe_2_O_4_ spheres are uniformly distributed on the rGO nanosheets. The HRTEM image shown in Figure 6c shows two planes with lattice spacing of ca. 0.25 and 0.21 nm, which correspond to the (311) and the (400) planes of the spinel ZnFe_2_O_4_ crystals. In addition, the angle of the two is measured as 25.24°, which is consistent with computations of the crystal structure. Moreover, the inserted fast Fourier transform (FFT) image reveals the typical hexagonal diffraction ring of rGO. As shown in Figure 6d, the selected area diffraction (SAED) pattern shows a series of distinct diffraction rings, which can be readily indexed to the (220), (311), (400), (422), (511) and (440) planes of the spinel ZnFe_2_O_4_ crystal, in line with the above XRD results. Moreover, the SAED pattern exhibits the polycrystalline nature of the 0.5 wt % ZnFe_2_O_4_/rGO composite. Furthermore, the composition of the sample was analyzed by high-angle annular dark-field imaging scanning transmission electron microscopy (HAADF-STEM) and EDS. The element mappings in Figure 6e reveal the existence of C, Zn, Fe and O in the 0.5 wt % ZnFe_2_O_4_/rGO spheres confirming the formation of a composite structure with hollow spheres of ZnFe_2_O_4_ distributed on the carbon matrix.

### Gas sensing performance

**Figure 7:**
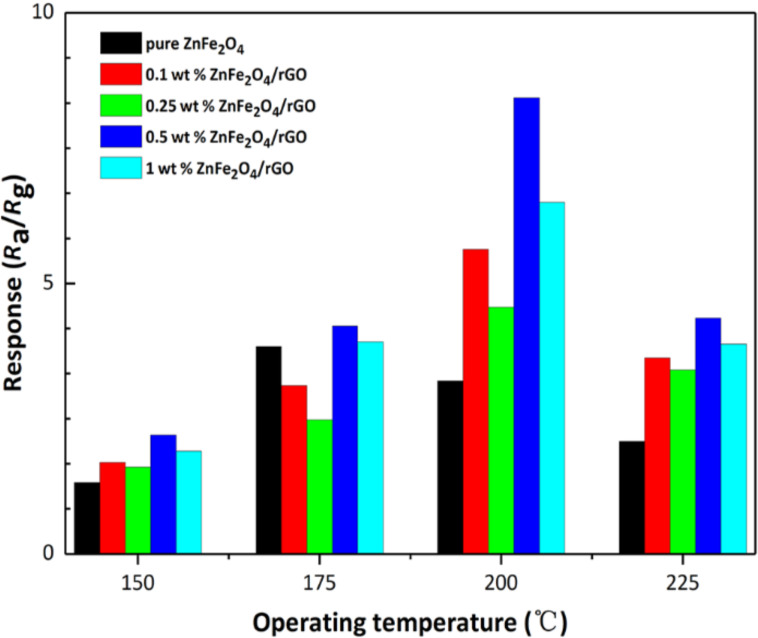
Response of the hollow spheres made of pure ZnFe_2_O_4_ and the four different ZnFe_2_O_4_/rGO composites used as gas sensors to 10 ppm acetone vapor at 150–225 °C.

The response of the fabricated gas sensors to 10 ppm acetone as a function of the operating temperature (150–225 °C) is shown in Figure 7. All the ZnFe_2_O_4_/rGO sensors show an enhanced response when the temperature is increased from 150 to 200 °C, while for the pure ZnFe_2_O_4_ sensor the response has a maximum at 175 °C. This tendency is ascribed to the higher surface activation energy at elevated temperature. As a result, the activation energy barrier of surface reactions with the target gas molecules is more easily overcome, resulting in an increased response [40–41]. At 200 °C, the 0.5 wt % ZnFe_2_O_4_/rGO sensor shows the highest response of 8.18. At a temperature of 225 °C, the responses decrease quickly, most likely due to the faster motion of the acetone molecules at elevated temperature. When moving faster, the adsorption of acetone at the sensor surface is less efficient, and the surface reaction of acetone and the chemisorbed oxygen ions is decelerated. Hence, the optimal operating temperature for the ZnFe_2_O_4_/rGO sensors is determined as 200 °C.

**Figure 8:**
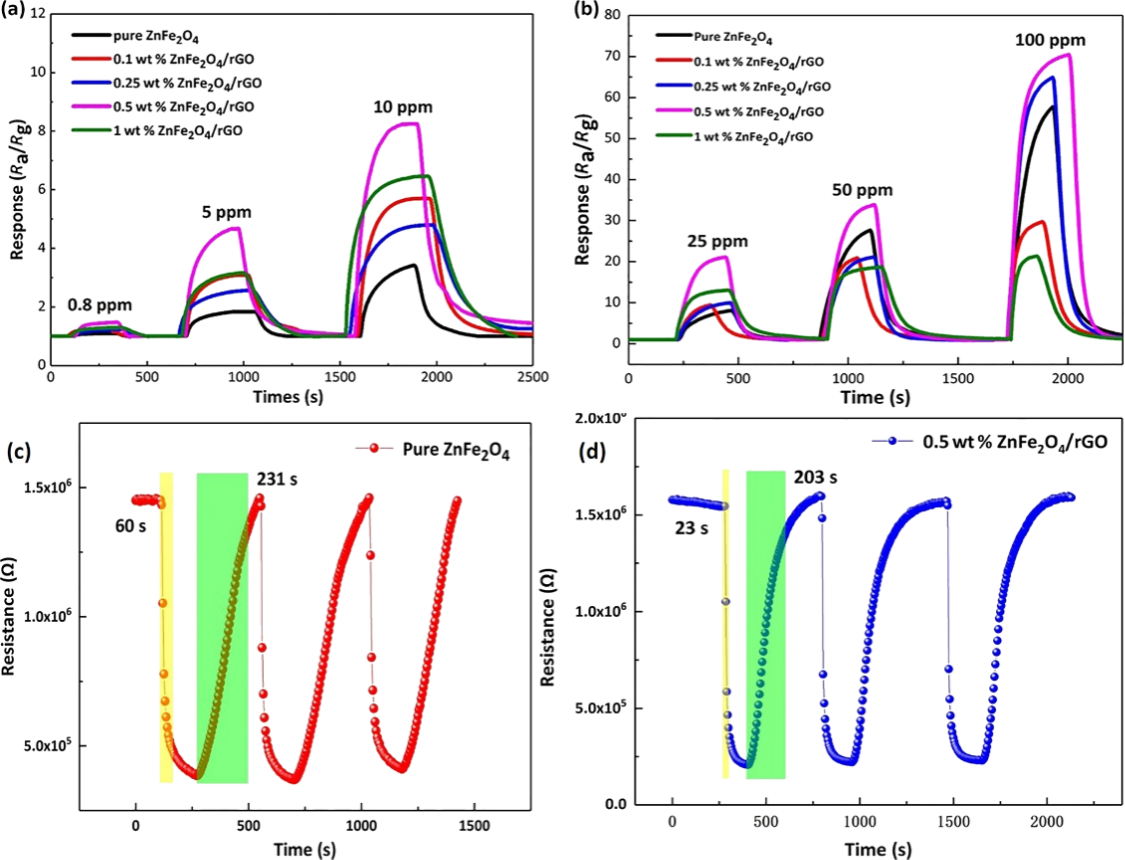
Dynamic response/recovery curves of the pure ZnFe_2_O_4_ and the four ZnFe_2_O_4_/rGO gas sensors to (a) 0.8–10 ppm and (b) 25–100 ppm acetone at 200 °C. (c, d) The short term stability and the response/recovery time of the pure ZnFe_2_O_4_ and the 0.5 wt % ZnFe_2_O_4_/rGO sensors to 10 ppm acetone measured in three cycles.

Figure 8a shows the dynamic response of the five sensors to 0.8–100 ppm acetone at 200 °C. It can be observed that the response increases significantly for the samples with higher content of rGO. Notably, the ZnFe_2_O_4_/rGO sensor containing 0.5 wt % of rGO showed an improved acetone sensing performance, the response to 0.8 and 10 ppm acetone was 1.50 and 8.18, while the corresponding values of the pure ZnFe_2_O_4_ sensor were 1.09 and 3.20. However, the response decreases with the increase of the rGO content from 0.5 to 1 wt %, which may be explained as follows. The addition of a large amount of the aqueous dispersion of GO to the organic solvent, in which the reaction occurrs, affects the formation of the ZnFe_2_O_4_ hollow spheres [17,39], resulting in a limitation of surface reactions. In addition, as shown in Figure 8b, the response of the sensors to 25–100 ppm acetone was also probed employing the same experimental conditions. The ZnFe_2_O_4_/rGO sensor with 0.5 wt % rGO still exhibits the best sensing performance. As shown in Figure 8c and Figure 8d, the sensor of pure ZnFe_2_O_4_ and the ZnFe_2_O_4_/rGO sensor with 0.5 wt % rGO both showed a good short term stability. Moreover, the 0.5 wt % ZnFe_2_O_4_/rGO sensor showed a shorter response/recovery time to 10 ppm acetone at 200 °C. The response time has been measured as 60 s for the pure ZnFe_2_O_4_ sensor and only 23 s for the 0.5 wt % ZnFe_2_O_4_/rGO sensor.

**Figure 9:**
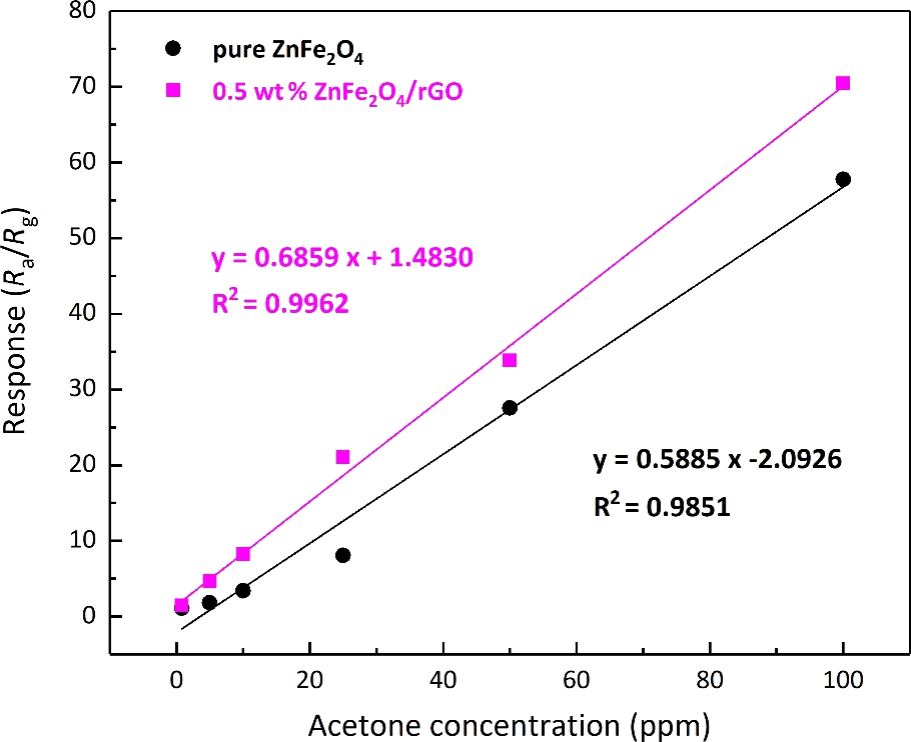
Linear response of (a) the hollow spheres of pure ZnFe_2_O_4_ and (b) of ZnFe_2_O_4_/rGO with 0.5 wt % rGO to acetone at different concentration.

Figure 9 shows the responses of the pure ZnFe_2_O_4_ sensor and the 0.5 wt % ZnFe_2_O_4_/rGO sensor to acetone at different concentration at 200 °C and the corresponding fitting data. The results demonstrate that the response of the hollow spheres made of pure ZnFe_2_O_4_ and of ZnFe_2_O_4_/rGO with 0.5 wt % rGO are more intense at higher concentration of acetone. Moreover, it is obvious that the linear response of the 0.5 wt % ZnFe_2_O_4_/rGO sensor has a steeper slope indicating an improved potential for quantitative gas analysis compared to the gas sensor of pure ZnFe_2_O_4_. For this composite gas sensor, the response to 1 ppm acetone is calculated to be 2.19, while the response of the pure ZnFe_2_O_4_ sensor to 5 ppm was only 1.83. Thus, the ZnFe_2_O_4_/rGO gas sensor with 0.5 wt % has a lower detection limit and shows an improved response. Furthermore, the 0.5 wt % ZnFe_2_O_4_/rGO gas sensor showed a better performance of acetone sensing at a lower temperature than any previously reported ZnFe_2_O_4_-based gas sensors (Table 1).

**Table 1:** Comparison of the acetone sensing performance of various reported sensors based on different materials.

Materials	Operating temperature (°C)	Conc. (ppm)	Response	LOD^a^ (ppm)	Ref.

NiO-ZnO hybrid	260	200	7.5	5	[41]
ZnFe_2_O_4_ nanosheet-assembled hollow spheres	215	20	9.8	1	[42]
ZnO/ZnFe_2_O_4_ hollow spheres	280	50	5.2	10	[43]
1 wt % rGO/α-Fe_2_O_3_	225	10	2.9	5	[25]
1 wt % RGO-CdFe_2_O_4_	270	10	20.7	0.01	[44]
0.125 wt % RGO/ZnFe_2_O_4_	275	10	4.0	1	[31]
ZnFe_2_O_4_-graphene quantum dots	room temperature	10	3.2	5	[32]
0.5 wt % ZnFe_2_O_4_/rGO hollow spheres	200	10	8.2	0.8	this work

^a^LOD represents the limit of detection.

**Figure 10:**
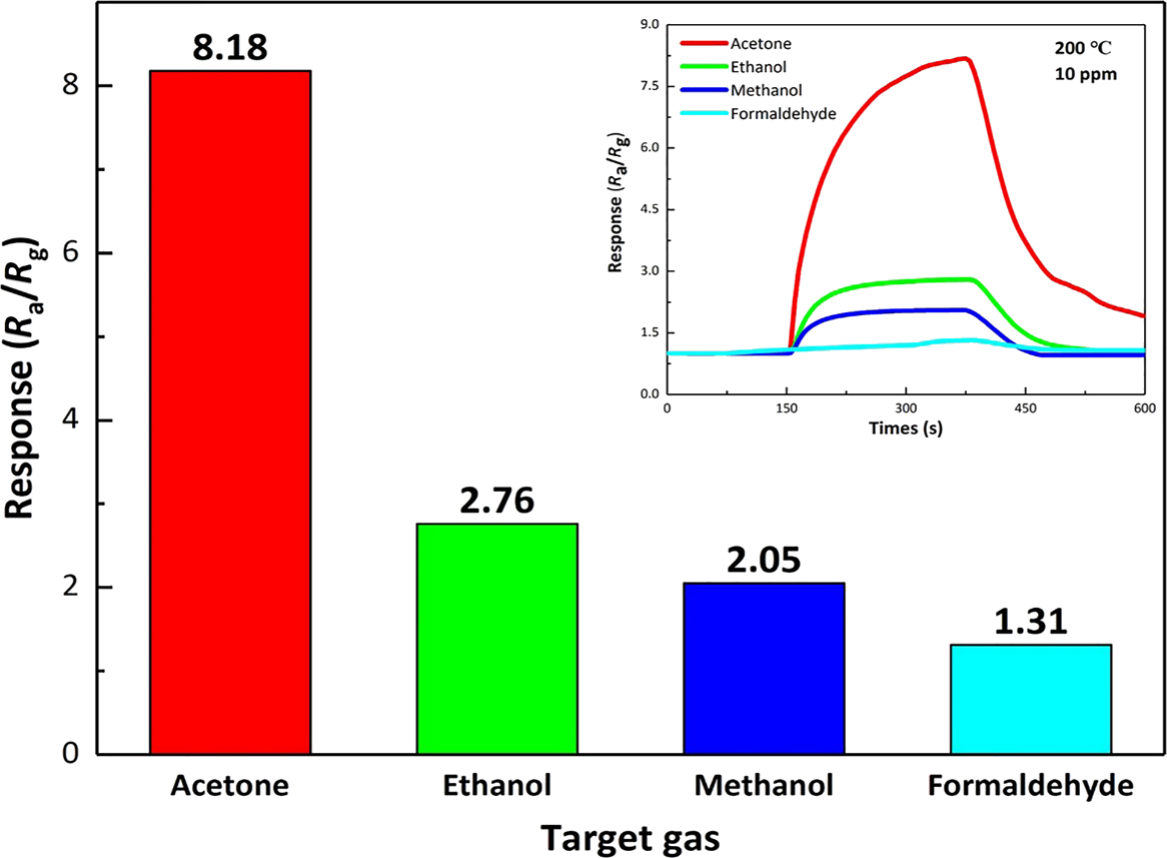
Response of the 0.5 wt % ZnFe_2_O_4_/rGO sensor to 10 ppm of acetone, ethanol, methanol and formaldehyde at 200 °C.

The selectivity of gas sensors is an important issue for practical applications. Herein, ethanol, methanol and formaldehyde were selected to measure the selectivity of the 0.5 wt % ZnFe_2_O_4_/rGO gas sensor at 200 °C. As shown in Figure 10, the responses to 10 ppm acetone, ethanol, methanol and formaldehyde were 8.18, 2.76, 2.05 and 1.31, respectively. It is obvious that the most intense response of the 0.5 wt % ZnFe_2_O_4_/rGO gas sensor corresponds to acetone. It is about three times more pronounced than the response to ethanol. The results reveal the excellent selectivity of the composite sensor for acetone. These findings are similar to the results of several previously reported works [45–47].

### Acetone sensing mechanism

The most accepted explanation of the acetone sensing mechanism of ZnFe_2_O_4_, which is an n-type semiconductor, is described as a reaction at the gas–solid interface. When the ZnFe_2_O_4_ gas sensor is placed in air at 200 °C, the adsorbed oxygen atoms capture free electrons from the conduction band to form adsorbed oxygen anions (O_2_^−^, O^−^). In ZnFe_2_O_4_, an electron depletion layer is left, which will result in an increase of the resistance. Upon exposure to acetone, the acetone molecules will adsorb to the surface of ZnFe_2_O_4_ and react with the adsorbed O_2_^−^ and O^−^ anions at the ZnFe_2_O_4_ grain boundaries. Subsequently, the electrons will be released back to the conduction band and the resistance will decrease again. The whole process can be described by Equations 2–5 [31,48].

[2]
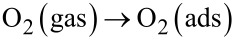


[3]
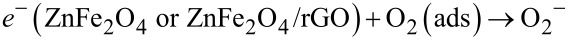


[4]
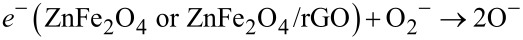


[5]
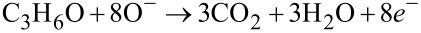


**Figure 11:**
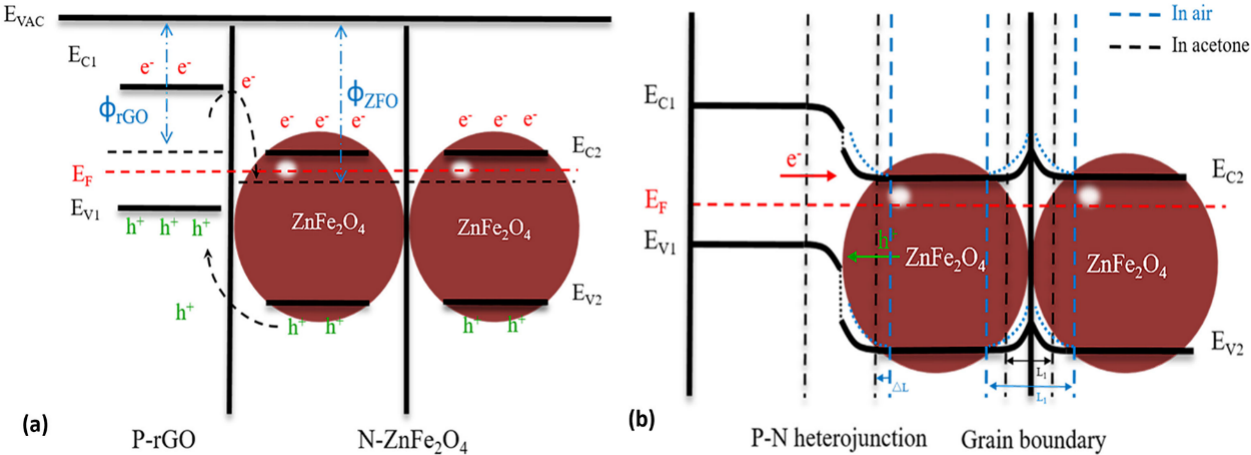
Schematic of the proposed acetone sensing mechanism of ZnFe_2_O_4_/rGO. Band diagram of rGO and ZnFe_2_O_4_ (a) at equilibrium condition (b) in air and in acetone.

In this work, the 0.5 wt % ZnFe_2_O_4_/rGO gas sensor showed an enhanced acetone sensing performance compared to the sensor of pure ZnFe_2_O_4_ due to the unique hollow structure of the ZnFe_2_O_4_ spheres and the excellent electrical properties of the 2D-rGO nanosheets [49–50]. More precisely, the enhanced acetone sensing properties may be attributed to several aspects. First, the sensing mechanism is based on the mentioned reaction at the gas–solid interface. Second, an appropriate content of rGO can improve the electron mobility inside the composites, which is helpful for the interface reaction [24,51]. Third, as shown in Figure 11a, the Fermi energy levels equalize when two semiconducting systems are in contact via the transfer of carriers. Consequently, heterojunctions and regions of electron depletion will be formed at the interface between rGO, which is a p-type semiconductor, and ZnFe_2_O_4_, an n-type semiconductor. In addition, there will also exist a potential barrier at the grain boundaries. In Figure 11b, when air is introduced, the resistance of the sensor will decrease as a result of the construction of the depletion layers and the potential barriers at the grain boundaries (see also Equations 2–5). When acetone is introduced, the resistance of the sensor will decrease again because of the contraction of the depletion layers and the potential barriers [19,25,52–53]. As a result, a sensing signal is obtained.

## Conclusion

In this work, the capability of different ZnFe_2_O_4_ sensors to detect acetone at low-ppm level was improved by incorporation of rGO. The rGO mass fraction of the ZnFe_2_O_4_/rGO spheres was 0, 0.1, 0.25, 0.5 and 1 wt %, respectively. Upon introduction of rGO, the size and the hollow structure of the sphere were not affected, while the surface morphology was modified and became more regular. The ZnFe_2_O_4_/rGO sensor containing 0.5 wt % rGO showed a sensitive linear response to a low concentration of acetone at 200 °C and exhibited a good selectivity. The response of the 0.5 wt % ZnFe_2_O_4_/rGO sensor to 10 ppm acetone was 8.18, which is about 2.5 times higher than that of the corresponding sensor made of pure ZnFe_2_O_4_. Therefore, this sensor shows great promise for detecting acetone at low concentration (ppm). It is a suitable candidate for the nondestructive diagnosis of diabetes by means of concentration measurements of exhaled acetone vapor if the sensitivity and the response speed to acetone at ppb-level are further improved.
